# Current treatment options and challenges in patients with Type 1 diabetes: Pharmacological, technical advances and future perspectives

**DOI:** 10.1007/s11154-021-09635-3

**Published:** 2021-03-23

**Authors:** Federico Boscari, Angelo Avogaro

**Affiliations:** Department of Medicine, Unit of Metabolic Diseases, University of Padova, Padova, Italy

**Keywords:** Type 1 diabetes, Technology, Artificial pancreas, Pancreas transplantation, Islet transplantation, Stem cells

## Abstract

Type 1 diabetes mellitus imposes a significant burden of complications and mortality, despite important advances in treatment: subjects affected by this disease have also a worse quality of life-related to disease management. To overcome these challenges, different new approaches have been proposed, such as new insulin formulations or innovative devices. The introduction of insulin pumps allows a more physiological insulin administration with a reduction of HbA1c level and hypoglycemic risk. New continuous glucose monitoring systems with better accuracy have allowed, not only better glucose control, but also the improvement of the quality of life. Integration of these devices with control algorithms brought to the creation of the first artificial pancreas, able to independently gain metabolic control without the risk of hypo- and hyperglycemic crisis. This approach has revolutionized the management of diabetes both in terms of quality of life and glucose control. However, complete independence from exogenous insulin will be obtained only by biological approaches that foresee the replacement of functional beta cells obtained from stem cells: this will be a major challenge but the biggest hope for the subjects with type 1 diabetes. In this review, we will outline the current scenario of innovative diabetes management both from a technological and biological point of view, and we will also forecast some cutting-edge approaches to reduce the challenges that hamper the definitive cure of diabetes.

## Introduction

Type 1 diabetes (T1D) is an autoimmune disease characterized by the disruption of pancreatic beta cells: this leads to a progressive reduction of insulin secretion and subsequent hyperglycemia, along with lipid and protein metabolism derangements. The DCCT-EDIC study showed that hyperglycemia, in type 1 diabetes, is associated with micro-and macrovascular complications and increased mortality[1–3]. To survive, subjects with type 1 diabetes must rely on exogenously injected insulin in subcutaneous tissue: this ensures adequate basal and prandial insulin concentrations to recreate physiological insulin profiles to avoid ketoacidosis and hyperglycemia-related complications [4]. The most relevant limiting factor for achieving good glycemic levels is hypoglycemia, defined as glycemic values lower than 70 mg/dl (3.9 mmol/L), determined by a discrepancy between insulin administration and carbohydrate (CHO) intake [5–8]. Hypoglycemia impacts the quality of life and leads to acute complications like seizures and coma, and, potentially, to a heart attack. The fear of hypoglycemia leads the patients to accept higher glycemic values, making more difficult the achievement of a good metabolic control [9–11]. To inject suitable insulin doses T1D subjects (T1Ds) must: 1. monitor their glucose values several times/day (self-monitoring of blood glucose, SMBG), 2. know the exact amount of CHO in their diet, 3. calculate the correct ratio between CHO taken and insulin to administer (I: CHO ratio), 4. estimate the impact of physical activity, illness, and stressful episodes. All these commitments lead T1Ds to face numerous daily decisions with an important deterioration of quality of life (QoL) [12–14]. Regrettably, the subcutaneous administration of insulin is non-physiological since the portal-to-periphery ratio of hormone concentrations is reversed leading to a relative peripheral overinsulinization and frequently unmatched insulin levels for the prevalent glucose concentrations. To overcome this problem, new insulin with more physiological pharmacodynamic have been introduced in the market; basal insulin analogs with longer duration (degludec, glargine U300), demonstrated their efficacy in maintaining metabolic control without hypoglycemia, especially during the night. [15–19]. On the other hand, new ultra-rapid prandial insulin analogs lead to better postprandial glycemic control reducing hyperglycemia in the early post-prandial phase. In a recent meta-analysis, faster aspart demonstrated efficacy in T1Ds in terms of reduction of HbA1c without increasing the overall hypoglycemic episodes. [20, 21]. Furthermore, insulin pumps (continuous subcutaneous insulin infusion, CSII) could ensure a more physiological approach [22]. Beyond insulin, other drugs have been proposed for the management of type 1 diabetes, in association with insulin [23]. In particular, sodium–glucose co-transporter-2 (SGLT2) inhibitors can reduce the HbA1c along with weight loss and reduction of daily insulin dose [24], especially in overweight subjects: this paved the way to the approval for dapagliflozin use in overweight (body mass index > 27 kg/m^2^) T1Ds in association with insulin in several countries. However, it is important to underline the potential risk of ketoacidosis associated with the use of these drugs, especially when the insulin dose is excessively down-titrated [25, 26]. In addition to SGLT2 inhibitors, other drugs approved in type 2 diabetes have been evaluated for T1Ds. Metformin demonstrated a reduction in BMI and insulin requirements, with no clear effects on HbA1c [27]. Glucagon-like peptide 1 receptor agonists (GLP-1RA), used for the treatment of T2D and obesity, demonstrated potential efficacy in clinical trials also in T1Ds when adjunct to insulin; a recent meta-analysis confirmed that GLP-1RA improve glycemic control, reduce severe hypoglycemia, body weight, and insulin requirements [28].

The monitoring of glucose levels ​​has also been improved with the introduction in the market of smaller, more accurate, glucose monitoring systems that allow patients with T1Ds to visualize every 1 to 5 min their glucose values [29]. Despite these innovations, people with type 1 diabetes still have a reduced life expectancy [30], with an increased risk of both macro-and microvascular complications and a worse quality of life compared to the non-diabetic population [31]. To optimize diabetes control, three main fields have been investigated: pharmacological, technological, and biological approaches. From a pharmacological standpoint, new insulin formulations have undoubtedly allowed higher efficacy, safety, and flexibility in the management of diabetes. The technological approach has allowed more sophisticated insulin pumps, sensors, glucometers, capable of simplifying, and improving diabetes management. Technology has also helped the management of diabetes thanks to easier data recording and safer data sharing between clinicians, patients, and caregivers. The biological approach aims to completely replace the production of insulin: in the last decades, either pancreas or beta-cell transplantation has dramatically improved as well as immunosuppression so that beta-cell replacement can now be considered an option to cure T1Ds. Regrettably, this type of approach is limited by the lack of organs and by the exposure of subjects to the consequence of immunosuppressive therapy, so that researchers are actively seeking to create new beta cell source from stem cells, to guarantee insulin production without the immunosuppressive therapy. This review describes innovative technological and biological approaches for diabetes management, highlighting future strategies that could be developed to reduce the burden related to diabetes and maybe to find a cure.

## Technology innovation

In recent years technology has revolutionized the management of diabetes: the technological approach is based on the use of insulin pumps and sensors for continuous glucose monitoring, and on the possibility to integrate these 2 systems to create a device capable of autonomously modifying the administration of insulin according to the values ​​detected by the sensor, thus creating the so-called artificial pancreas or closed-loop system.

## State of the art

### Insulin pump

Since their introduction in the 70 s, these devices have undergone important improvement, both in terms of portability and functionality. Insulin pumps allow the continuous administration of rapid insulin analogs, infused at different pre-programmable basal rates that mimick the secretion of physiological hormone response. Furthermore, the administration of meal insulin boluses can also be protracted to allow a better insulinization in response to meals enriched in protein and fat that have a significantly slower absorption. CSII leads to an improvement in glycemic control and reduction of hypoglycemia. Several studies demonstrated a statistically significant reduction of both HBA1c and hypoglycemic events in patients on CSII (Table 1) [32–38]. In a meta-analysis of the available randomized controlled studies (RCT), Pickup and colleagues showed that CSII reduces HBA1c by 0.21% as compared to multiple daily injection (MDI) therapy [33]. Similarly, in 2010 Monami and colleagues reported a reduction of HbA1c of 0.3% [34]. All meta-analysis compared CSII efficacy vs glargine or NPH insulin basal but relatively fewer data are available on CSII efficacy vs. MDI performed with new basal analogs. However, a more recent meta-analysis demonstrated superior efficacy of CSII in reducing HbA1c also in trials in which a rapid-acting analog was used; the advantage of CSII vs. MDI was smaller than that observed in trials using regular human insulin [35]. Data on hypoglycemic events are less clear: a similar hypoglycemic risk between CSII and MDI has been reported. Notably, it must be also acknowledged that there are insufficient data about efficacy in children [36]. CSII requires greater management skills and commitment than MDI therapy but, at the same time, allows greater flexibility in controlling the daily activities, and this leads to an improvement of the patients’ quality of life. Several trials demonstrated a better acceptance of this approach with a parallel reduction of the burden related to diabetes [39, 40]. The risks associated with ketoacidosis secondary to the occlusion of the infusion set were reported to be minimal [41].

**Table 1:** Summary of meta-analysis that evaluated CSII efficacy vs MDI

Meta-Analysis	Population	Number of studies considered	MDI Therapy	Effects on HbA1c	Effects on Hypoglycemia	Comments
2008 Pickup JC, Sutton AJ [33]	Adults and Children	22 (10 in children, 12 in adults)	isophane- or lente-type intermediate-acting insulin in combination with regular or monomeric insulin at meals	-0.21% (95% CI: 0.13–0.30%) Improvement of HBA1c was greater in those with the highest HbA1c values on MDI	Reduction of severe hypoglycemia during CSII (RR 2.89, from 1.45 to 5.76) Hypoglycemia reduction was greater in those with most severe hypoglycemia on MDI	
2008 Jeitler K, Horvath K, Berghold A, Gratzer TW, Neeser K, Pieber TR, Siebenhofer A [37]	Adults	17 RCT	NPH-glargine Regular-Rapid analogs	− 0.4 (95% CI: − 0.82, − 0.01, p < 0.001)	No differences in severe Hypoglycemia	Total daily insulin requirements were lower with CSII than with MDI therapy
2010 Misso ML, Egberts KJ, Page M, O'Connor D, Shaw J [38]	Adults and children	23 RCT		-0.3% (95% CI: -0.4, -0.1, p value = 0.001	Reduction of severe hypoglycemia	Reduction of daily insulin requirement in CSII (-7 U, 95% CI -11 to -3) CSII was preferred for treatment satisfaction and quality of life (different scales used)
2010 Monami M, Lamanna C, Marchionni N, Mannucci E [34]	Adults and children	11 RCT	NPH-glargine Rapid analogs	-0.3 (95% CI -0.4; -0.1, p < 0.001) Reduction of HbA1c wasn’t significant in trials enrolling subjects < 10 years	No differences in severe hypoglycemia	
2017 Benkhadra K, Alahdab F, Tamhane SU, McCoy RG, Prokop LJ, Murad MH [32]	Adults and children	25 RCT	NPH-glargine Regular-Rapid analogs	- 0.37 (95% CI: -0.24, –0.51, p > 0.001) Adults -0.42 (95% CI: -0.23, -0.61; P = 0.001) Children -0.32 (95% CI: -0.13, -0.51; P = 0.002)	No differences in severe hypoglycemia	
2018 Qin Y, Yang LH, Huang XL, Chen XH, Yao H [36]	Children	8 RCT	NPH-glargine Regular-Rapid analogs	-0.25 (95% CI: -0.43, -0.07, p = 0.007)	No differences in severe hypoglycemia	Similar total daily insulin doses between CSII and MDI, reducted after long-term (12 months) Similar incidence of ketoacidosis
2019 Pala L, Dicembrini I, Mannucci E [35]	Adults and children	40 RCT	NPH-glargine Regular-Rapid analogs	HbA1c reduction with rapid analogs was smaller than in trials with regular human insulin (HbA1c difference: − 0.29 (95% CI -0.46, -0.13) vs − 1.93(95%CI: -1.84, -0.42), p = 0.02) HbA1c reduction was similar with NPH or long-acting analogs as basal insulin in the control groups		CSII was associated with a significant increase in the incidence of reported diabetic ketoacidosis (DKA) in trials comparing CSII with conventional insulin therapy, with no differences in comparisons with basal-bolus

*RCT* randomized crossover trials, *NPH* neutral protamine Hagedorn insulin, *CI* confidence interval

Last but not least, CSII-based treatment is associated with a reduction of mortality and complications [42], as reported by the Swedish register that evaluated more than 18,000 T1Ds treated with CSII or MDI [43]. Reduction of mortality, especially related to cardiovascular events, could be related to lower hypoglycemic events and more stable glucose values.

Another approach for insulin infusion is represented by continuous intraperitoneal insulin infusion (CIPII). CIPII provides an alternative insulin administration, through an implantable pump, allowing a more physiological delivery since insulin is absorbed through the portal system, thus mimicking the physiological condition [44]. The need for surgery and the costs limit this option for T1Ds who fail to achieve satisfactory glycemic control with other treatments. Several studies demonstrated CIPII efficacy when compared to CSII in term of HbA1c and severe hypoglycemia reduction and treatment satisfaction [45, 46]

### Continuous glucose monitoring (CGM)

Continuous glucose monitoring (CGM) represents an awesome improvement in the possibilities of monitoring the glucose levels: these devices continuously detect the glucose concentrations in subcutaneous tissue thanks to small sensors that can be replaced every 7–14 days. CGM systems can be divided into real-time (rt-CGM) and intermittently scanned (is-CGM) devices. Rt-CGM provides real-time glucose values, ​allowing the patient to view, not only the glucose levels but also the future trends prediction and past trends on both the receiver or on a smartphone app which provides appropriate alerts for both high and low glucose readings: with rt-CGM the patient is aware when a given glycemic threshold is exceeded or when it is about to be exceeded [47–49]. These devices demonstrated superiority in their efficacy over SMBG in terms of HbA1c reduction, glucose variability, and hypoglycemia reduction in subjects treated either with CSII or MDI [50–61], as shown in Table 2. Unfortunately, their use may be intermittent for weekly sensor replacement [62]. Over the last years, their accuracy has been improved, and some of them have been approved for non-adjunctive use, allowing patients with T1D to adopt decisions regarding their insulin therapy without the need for capillary glucose control [63]. Some devices need calibration vs. capillary glucose to ensure adequate accuracy, but now devices factory-calibrated are available [64]. An implantable subcutaneous sensor of 180 days duration has been recently introduced: this approach avoids the need for weekly sensor replacement with similar efficacy in terms of metabolic control [65, 66]. Is-CGM or flash glucose monitoring system (FGM), on the other hand, does not provide alarms and allows the patient to view glycemic values and trends when the patient scans the sensor through the reader or mobile phone. Is-CGM has proven its effectiveness in improving glycemic control and reducing hypoglycemic risk [67–69]. Recently, a new version of is-CGM provided optional alerts for high and/or low glucose levels, thus advising T1Ds to perform a scan to evaluate the actual glucose level. All these devices lead to an improvement in QoL when compared to SMBG [52, 70, 71], due to the possibility to visualize data continuously without the need for finger sticks.

**Table 2:** Summary of meta-analysis that evaluated CGM and FGM efficacy vs SMBG

Meta-Analysis	Population	Number of studies considered	Effects on HbA1c	Effects on Hypoglycemia	Comments
2008 Golicki DT, Golicka D, Groele L, Pankowska E [54]	Children, CGM	5	− 0.02% (95% CI − 0.29 to 0.25; p = 0.87)	No differences	increase in the number of insulin dose changes
2008 Chetty VT, Almulla A, Odueyungbo A, Thabane L [55]	Adults and children, CGM	7	Non-significant reduction in HBA1c (0.22%; 95% CI:- 0.439% to 0.004%,p = 0.055	indication of decreased nocturnal hypoglycemia	
2012 Szypowska A, Ramotowska A, Dzygalo K, Golicki D [56]	Adults and children, CGM	7	-0.25; (95% CI from -0.34 to -0.17; p < 0.001)	No differences	inverse correlation between the HbA1c level and the frequency of sensor use
2012 Floyd B, Chandra P, Hall S, Phillips C, Alema-Mensah E, Strayhorn G, Ofili EO, Umpierrez GE [57]	Adults and children, CGM	14	-0.3% (95% CI from 0.4 to -0.2), p < 0.0001	Shorter duration of hypoglycemia (75 ± 39 versus 89 ± 19 min/day), reduction of hypoglycemia duration of -15.2 min/day, p < 0.0001	Shorter duration of hyperglycemia (172 ± 125 versus 217 ± 152 min/day, p = 0.04)
2012 Yeh HC, Brown TT, Maruthur N, Ranasinghe P, Berger Z, Suh YD, Wilson LM, Haberl EB, Brick J, Bass EB, Golden SH [58]	Adults and children, CGM	8	Significative HbA1c reduction of 0.26% [95% CI, 0.33% to 0.19%]), sensor adherence associated with HbA1c level reduction	No differences in severe hypoglycemia	Reduction in time spent in the hyperglycemic range
2012 Langendam M, Luijf YM, Hooft L, Devries JH, Mudde AH, Scholten RJ [59]	Adults and children, CGM	22	CSII: -0.7%( 95% CI -0.8% to - 0.5%) MDI: -0.2%, (95% CI -0.4% to -0.1%)	No differences	
2013 Poolsup N, Suksomboon N, Kyaw AM [60]	Children, CGM	10	– 0.13% (95% CI -0.38% to 0.11%, p = 0.27)	No differences	
2017 Benkhadra K, Alahdab F, Tamhane S, Wang Z, Prokop LJ, Hirsch IB, Raccah D, Riveline JP, Kordonouri O, Murad MH [61]	Adults and children, CGM	11	-0,276 (95% CI -0.465 to -0.087 Stratified analysis by age results was statistically significant only in the age groups of > 15 years	No difference in time spent in hypoglycemia and number of hypoglycemic events	
2020 Gordon I, Rutherford C, Makarounas-Kirchmann K, Kirchmann M [69]	Adults and children, FGM	34	-0.41% ([95% CI -0.51%, -0.31%]; P < 0.001		

*CI* confidence interval

Both Rt-CGM and is-CGM provide predictions of the glucose levels based on previous glucose readings: these data could be used by T1Ds to adjust insulin correction or prandial boluses and CHO intake. This represents additional support in the management of T1DM, in particular at mealtime, when multiple parameters such as insulin: carbohydrate ratio, glucose target, and correction factor should be taken into account. Several recommendations have been published regarding trend arrow management: as an example, a percentage or fixed values could be added or subtracted to a prandial insulin bolus based on the rate of glucose changes [72, 73]. Recently, more personalized approaches have been introduced based on insulin sensitivity factors and different baseline glucose levels. [74, 75]

The availability of these devices has changed the metric to assess glucose control: the possibility to visualize daily glucose profiles have shifted the gold standard parameter for metabolic control HBA1c to parameters such as time in target range (TIR), time spent between 70 and 180 mg/dl, which have updated the goals to be achieved by the patients [76]. Other parameters complementary to TIR are time spent with glucose values below 70 mg/dl, time below range (TBR), and time spent above 180 mg/dl, time above range (TAR). These parameters have some limits, related to the lack of an established standard for glucose measurement with CGM: as suggested by several authors, TIR should be regarded as a complement to HbA1c [77]. Indeed HbA1c values have been considered over the last decades the parameter that better correlates with clinical outcomes, even though additional evidence of a correlation between TIR and diabetes complications are emerging, both for micro and macrovascular complications [78–80].

### Blood glucose meters (BGM)

Although the use of CGM is increasing, some T1Ds continue to use BGM to check their glucose values: it might be related to either the lack of CGM accuracy, or their cost and unacceptability [81, 82]. Several studies demonstrated the efficacy of BGM in reducing both HBA1c and the hypoglycemic events when tests are performed correctly, usually from six to ten times a day, even if the visualization of the glucose levels are intermittent [83]. BGM technology have been improved over the last years [84]. Accuracy of devices is crucial not only to correctly manage the disease but also to calibrate CGM; accuracy of BGM could be compared to the reference values of venous blood glucose [85]. New BGM could be connected to a smartphone app leading to a better patient’s engagement and to the possibility of sharing directly data with phisicians or caregivers [86]. Several devices also have other features, such an alarm to remind the subject to check her/his blood sugar, or a bolus calculator integrated into the BGM that simplifies the calculation of prandial bolus amount based on the subjects’ parameter [87].

### Sensor augmented pump and first automatic systems

Given the superiority of the CSII over MDI and CGM over SMBG, the gold standard for the treatment of type 1 diabetes should be the combination of CSII with CGM, called Sensor Augmented Pump (SAP) Therapy. This combination is superior when compared to CSII + SMBG in terms of improving glycemic control and reducing hypoglycemia [51]. Nonetheless, subjects on SAP therapy in apparently good metabolic control spend several hours in both hypo and hyperglycemia, indicating that more precise approaches are required to obtain glycemic values ​​comparable to those observed in subjects without diabetes [88]. For this reason, systems with automated modification in insulin administration based on the values ​​detected by the sensor have been assessed. The first automated approach was dedicated to control hypoglycemia: the Low Glucose Suspend system (LGS) interrupts insulin infusion for a maximum of 2 h when a predetermined low glucose level is reached: this approach can reduce severe hypoglycemia, even if compared to SAP without LGS [89], especially in T1D at high risk of hypoglycemia or with reduced hypoglycemia awareness [90]. The second approach, a further step towards better management of diabetes, was achieved through the introduction of Predictive Low Glucose suspend (PLGS), capable of suspending the basal administration of insulin when hypoglycemia was predicted by the sensor with a further reduction of hypoglycemic risk [91–93]. In this context, real-life studies have shown the efficacy of this algorithm in improving metabolic control [94–96].

### Artificial pancreas

**Fig. 1:**
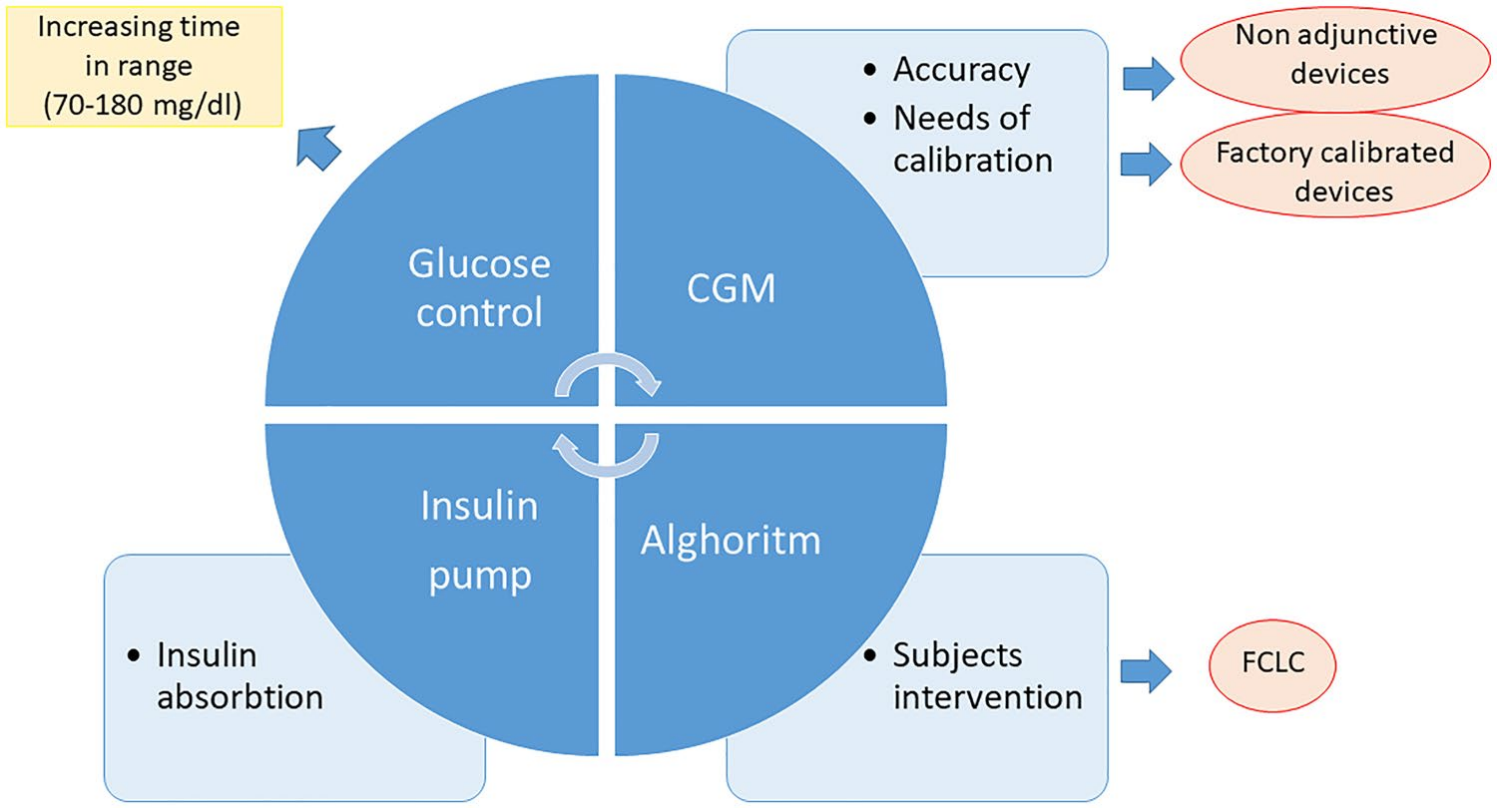
Artificial pancreas components, its limitations, and future perspectives: The algorithm modifies automatic insulin infusion throughout the insulin pump based on glucose values registered by CGM to optimize glucose control increasing time spent in a target (70–180 mg/dl). Challenges are related to insulin absorption that should be accelerated, CGM accuracy, and the need for calibration. To reduce the burden related to diabetes a full closed-loop control that minimizes the subject's intervention could completely automatize insulin therapy. FLCL: full closed-loop control, CGM: continuous glucose monitoring

The artificial pancreas (AP) or closed-loop control (CLC) system is a technology that allows the control of blood glucose concentrations in a completely automated manner. This device is comprised of an insulin pump, a CGM, and a control algorithm (CA) that automatically modifies insulin infusion according to prevailing glucose concentrations. Insulin infusion is therefore modified every few minutes based on new glucose values received by CGM: CLC increases insulin infusion when glucose values are increasing and decreases or suspends insulin infusion in case of significant reduction of glucose levels to minimize the risk of hypoglycemia (Fig. 1). Different models have been developed with different insulin pumps and different CGM and especially different CA, the “brain” of the system [97, 98]. In the last decade, several studies have assessed AP performances, initially in the inpatient setting [99, 100] to evaluate its safety and efficacy, then in patients’ real-life conditions to demonstrate their feasibility [101, 102]. All these trials established the superiority of AP compared to CSII or SAP, in terms of time spent in target, hypoglycemia reduction, HbA1c improvements, and acceptability by T1D subjects. Performances of AP were evaluated also in children and adolescents [103] and in pregnant women with T1D [104–106].

These trials lead to the introduction of the first commercially available CLC system, MiniMed 670G (Medtronic MiniMed, Inc., Northridge, CA, USA): this device is called a Hybrid closed-loop (HCL) because subjects have to announce meal intake to avoid postprandial hyperglycemia [107, 108]. In a pivotal registration trial this device showed, in both adults and adolescents, its efficacy [109] with a reduction in HBA1c values (from 7.7% ± 0.8% to 7.1% ± 0.6% (P < 0.001) in adolescents, and from 7.3% ± 0.9% to 6.8% ± 0.6% (P < 0.001) in adults, and with a parallel increase of TIR (from 60.4% ± 10.9% to 67.2% ± 8.2% (P < 0.001) in adolescents and from 68.8% ± 11.9% to 73.8% ± 8.4% (P < 0.001) in adults. Similar results were confirmed also in the pediatric population from 7 to 13 years [110], which often has a more challenging glycemic control. The efficacy of the system is proportional to the time when CLC is active (auto mode) [111]. Recently, a randomized controlled trial [112] confirmed the efficacy of Minimed 670G during 26 weeks, with a reduction of HbA1c and an increase of the time spent in target when compared to standard therapy. In this trial, it has been demonstrated also an improvement of diabetes-specific quality of life, evaluated through validated questionnaires. Currently, Minimed 670G is approved for T1Ds older than 7 years old. Since it has been commercialized in 2017 in the US, real-world data have been published [113], confirming the efficacy of the device. Nevertheless, in 1 year follow up observational study of T1Ds who started 670G use, a reduction of Auto Mode over time was observed; 46% of users stopped auto mode after 1 year and only 32% of subjects have used auto mode for at least 70% of the time [114]. These data suggest that beyond the efficacy of the device, other details have to be considered: suspension of auto mode was related to alarms reported by devices and the need for sensor calibration. Other reasons are related to the unique glucose target available (120 mg/dl), not acceptable by subjects with tighter glycemic control, such as during pregnancy. Factory calibrated CGM could solve the glitches related to calibration but improvements in AP models are necessary to increase their time of use. For this reason, an enhanced version of 670G, called 780G, obtained the CE mark in June; this new version could dispense automated correction boluses, have different optional glucose targets, and other features to increase the utilization of Auto Mode [115, 116].

Other devices have been evaluated and authorized for commercial use such as Tandem Control IQ, which proved its efficacy with a sensor that needs no calibrations, by increasing time in the target (from 61 ± 17% at baseline to 71 ± 12% at the end of 6 months study period), by reducing HBA1c values (-0.33% in CLC group) and hypoglycemic events [117]. This AP model was also assessed in the pediatric population [118] during a winter camp and showed its efficacy also in this specific population and during physical activity (percent time within range was 66.4 ± 16.4 vs 53.9 ± 24.8% with P-value 0.01). Since this system is available in the US from the beginning of 2020, the first real-life data have been published, confirming results obtained in clinical trials with improvements also in psychosocial outcomes [119].

Other HCL systems either received or are waiting for approval, and will be commercialized in the next years. CamAPS FX, which uses an algorithm non installed on an insulin pump but on a smartphone that communicates with the pump and sensor, received a CE mark for 1 year, and different trials demonstrated its efficacy also in adolescents and children [120, 121]. Diabeloop algorithm is also installed in a smartphone, and communicate with CGM and patch pump, CSII system without a catheter. In a randomized crossover trial, an increase of 9.2% of the time spent in the target was observed using this AP [122], and performances were evaluated also in more challenging situations as meals and physical exercise [123]. The Omnipod Horizon system [124] uses a patch pump and both its safety and efficacy were demonstrated in both adults and pediatric T1Ds even also in an outpatient setting [125, 126]. CLCs equally allow a better QoL, by reducing the burden related to diabetes by demonstrating a significant reduction of the time spent in diabetes management [127, 128]. These results need to be confirmed in real life since the effectiveness of clinical trials in selected subjects could have impacted the results. Since 2013 it is active as a movement for the development of open-source diabetes management systems (Do-It-Yourself Artificial Pancreas Systems, DIY), with the scope of accelerating AP development and access. This group aims to create an “open source” artificial pancreas, sharing algorithms with personalized settings, and glucose targets. These algorithms can communicate with several existing devices via Bluetooth thus enabling the conception of personalized insulin pumps and CGM, thus overcoming the marked systems. There are no clinical trials that have tested these systems, but data set analysis and real-world data suggested an improvement in HBA1c values and time spent in target and amelioration of QoL [129]. The lack of evidence by RCT and the absence of regulation poses also obvious legal problems for users.

### Further role of technology

Technology can simplify the management of diabetes: as an example, smart insulin pens with memory functions could record the insulin doses administered and transfer data via Bluetooth to dedicated apps [130]. Several smartphone apps for diabetes management have been developed, with the aim of help T1Ds to calculate insulin bolus, registered glucose data, track carbohydrate intake, or physical activity, with the possibility of sharing data on glycemic trends with clinicians. Also, CGM data could be managed with a smartphone app and shared in a cloud system, thus allowing also clinicians to visualize glucose values. This leads to the development of telemedicine methods which are tremendously useful when subjects can’t access the clinic, as recently occurred during Covid 19 pandemic [131, 132]. Similarly, data could be shared between T1Ds and caregivers, especially for example for children with T1D.

### Future prospectives

#### Continuous glucose monitoring

Even if substantial advancements have been made in the field of glucose sensors in terms of accuracy and portability, they remain needle-based device with reduced acceptance, especially in childhood. For this reason, researchers are working on new projects based on non-invasive glucose monitoring using alternative body fluids [133]. For example, a wearable patch to measure glucose on sweat has been tested [134], even if the contamination of skin, the impact of physical activities, and related changes in sweat production may represent major problems to solve. The determination of glucose in tears has also be considered using a contact lens-based system [135]: this device appears to have an accuracy comparable to the commercialized CGM system. Also, salivary glucose concentration correlates with those in plasma [136], but challenges related to the interferences with food or bacteria in the mouth limit the development of these devices.

#### Artificial pancreas

The real-life data obtained during the first-year experience with 670G [114] suggest that, beyond the efficacy in glucose control, other features should be considered to optimize automatic system use. The possibility to rely on correction boluses and the reduction of alarms in the 780G model and factory calibrated devices (in the AP model that uses non-adjunctive sensors) could improve device acceptance. Future prospective in AP development foresees the possibility of creating a full CLC (FCLC) that does not need a subject’s interventions. The main challenges in FCLC development are related to the difficulties in managing postprandial control with no meal announcement and during physical activity. In 2008 Weinzimer and colleagues [137] compared an FCLC and an HCL in an inpatient setting in adolescents. They demonstrated that, although the 2 systems performed similarly in the overnight period, the postprandial phase was better managed by HCL with meal announcement and prandial bolus administration 15 min before a meal with a postprandial peak of 226 ± 51 mg/dl vs 194 ± 47 (p-value 0.04). Similar results were obtained in an inpatient setting by Forlenza et al. in both adults and adolescents who underwent AP session with announced and unannounced meals; They showed that the postprandial CGM average was significantly lower for announced than for unannounced meals (140.6 ± 35.0 vs. 197.8 ± 44.1 mg/dl, p < 0.001) [138]. Challenges in postprandial peak management with no meal announcement are related to relative delay in insulin absorption. No significant improvements were observed in the postprandial phase in FCLC using FasterAspart compared to AspArt [139], thus demonstrating that the insulin absorption limits the postprandial peak management in FCLC. It has been shown that intraperitoneal insulin infusion allowed better control in unannounced meals, with a reduction of time spent in hyperglycemia in the postprandial phase [140]. This approach is not feasible in the real life in the majority of T1Ds but suggests that a more physiological and rapid insulin administration may be a potential solution for the postprandial peak challenge. Another approach to control postprandial peak is the pramlintide association. Pramlintide is an analog of amylin, co-secreted with insulin and deficient in T1D, that delays gastric emptying and suppresses glucagon secretion. Use of subcutaneous Pramlintide in FCLC was associated with a reduction of the postprandial magnitude of glycemic excursion (88 ± 42 vs. 113 ± 32 mg/dL; P = 0.006) compared with CLC alone [141]. Another challenge is related to physical activity management [142]: with commercialized AP models, the strategy of establishing a pre-set of different higher glucose targets reduces the risk of hypoglycemia. Methods to communicate physical activity to algorithms have been investigated such as adding hearth rate signal, as a surrogate of physical activity, measured through a heart rate monitor [143]. This approach reduced hypoglycemic risk during exercise and increased time in the target range (81% vs. 75%;P = 0.2). The necessity to wear another device limits this approach in real life.

#### Bihormonal artificial pancreas

The main limitation to achieve better glycemic control is the hypoglycemic risk: to overcome this problem, a bihormonal approach could maximize the efficacy of AP in reducing the risk of hypoglycemia thanks to the co-administration of glucagon. A bihormonal pancreas (BP) is similar to AP and consists of a CA installed in a smartphone that communicates with CGM, insulin, and glucagon pump. Studies that evaluated the efficacy of BP had a rather shorter duration as compared to studies that assessed AP; nonetheless, BP [144, 145] showed both safety and feasibility in in- and outpatients. The device has been also assessed in a randomized crossover trial conducted at home for 11 days and allowed a reduction in mean glucose levels and in time spent in hypo [146]. Notably, there were no physical activity limitations and the patients didn’t have to input the correct amount of CHO at each meal but just the meal size. A comparison between AP and BP was performed by Haidar, who showed an improvement of glucose control during BP use in an overnight period in both children and adolescents, with less strong evidence in real life in adults [147]. BP has some limitations related to the necessity of wear 2 insulin pumps and a lack of evidence of long-term effects of the continuous administration of subcutaneous glucagon. To minimize the impact of wearing 2 different pumps, a single wearable device integrating all components into one single device much more manageable in the real-life has been developed [148]. As shown in Table 3, some meta-analyses evaluated the efficacy of CLC in the outpatient setting, comparing different AP models with standard therapy (SAP). For example, Weisman in the first meta-analyses about AP efficacy reported that time in the target was 12·59% higher with artificial pancreas systems (p < 0·0001), and BP was associated with a greater improvement in time in the target and a reduction of time spent in hypoglycemia. [102]. A second meta-analysis performed by Bekiari and colleagues in 2018 confirmed these data[101], both overnight and over 24 h, and AP efficacy was confirmed even considering the pediatric population, separately [103].

## Biological approach

The definitive cure of diabetes may probably come from the biological approaches since they aim to replace the secretion of insulin indefinitely.

### State of art

The treatment of diabetes with exogenous insulin is often problematic due to recurrent hyper-and hypoglycemic episodes. In selected patients requiring a kidney transplant or suffering from recurrent severe hypoglycemia despite optimal medical therapy, pancreas or isolated islet transplantation can restore normal glucose metabolism.

#### Pancreas transplantation

Pancreas transplantation (PT) is an option for selected subjects: however, it requires major abdominal surgery. PT demonstrated its efficacy in restore normoglycemia, stabilize complications, and reduce the burden of hypoglycemia [149]. This approach is often contemplated in subjects who required previous/simultaneous kidney transplant for end-stage kidney disease since these subjects already require major surgery and immunosuppression: this strategy accounts for the majority of PT. New immunosuppressant agents have improved organ survival with a 5 years organ survival rate between 55 and 70%. The survival rate is increased when the pancreas is transplanted simultaneously to the kidney [150]. However, surgical intervention and immunosuppression effects, limit this option to a relatively small number of subjects.

#### Islet transplantation

Islet Transplantation (IT) has been introduced 20 years ago: this procedure is more acceptable by T1Ds since it is less invasive and repeatable and could be proposed to patients who are ineligible for PT. Islets are isolated from donor pancreases, purified, injected into the portal vein to obtain their engraftment in the liver [151]. When compared to optimal insulin therapy, IT demonstrated higher efficacy in reducing severe hypoglycemia [152] and preventing microvascular complications [153]. In medium long-term efficacy, IT is lower than that of PT in providing insulin independence with approximately 50% of patients remained insulin-independent at 5 years [154]. Similar to PT, these approaches are limited by the number of donor organs and by the need for immunosuppression. Encapsulation of islet has been evaluated, to prevent rejection and immune response. The presence of encapsulation creates a barrier that prevents the access of immune cells, thus limiting the necessity of immunosuppression, but also precluding optimal vascularization. Results were not satisfactory in terms of c peptide production and metabolic control but lead to a new strategy to protect transplanted cells [155].

## Future perspectives

### Beta cells replacement

PT and IT limits have sparked research for alternative sources of beta cells, potentially unlimited, and without the need for immunosuppression. Xenotransplantation represents a possible solution to the donor shortage and recent research in genetic modification and immunosuppressive regimens have increased interest in this area. Until now small clinical studies have considered this possibility, especially using pigs beta cells. Major barriers to xenotransplantation are represented by an instant blood-mediated inflammatory reaction, chronic rejection, and the risk of transmission of porcine infectious diseases. To overcome the risk of acute rejection, related to different cell surface epitopes between humans and donor, genetically modified Xeno islet have been created, using gene-editing techniques to alter proteins in cells surface. As demonstrated for human beta cell transplantation, encapsulation could protect also Xeno islet from immune attack. There are persistent barriers to xenotransplantation and further data are necessary to establish an ideal genetically modified porcine islet to evaluate the possibility of clinical studies [156].

Future strategies for beta-cell replacements are based on stem cell (SC) to create insulin-producing cells (IPC) from SC [157–161]. The first problem to solve is the source of IPC: these cells could be produced from a stem cell-derived from human embryonic cells (EC) [162] or human-induced pluripotent stem cells (IPSC) [163]. EC are pluripotent cells derived from the blastocyst that can proliferate indefinitely, and differentiate in different tissues. At variance, IPSC is derived from adult mature tissue, and is re-programmed by appropriate stimuli to pluripotent cells [164]. These types of stem cells are similar in pluripotent capacity, and have the potential to create IPC: however, there are significant ethical issues in using EC so that the main source of stem cells can be considered the IPSC. Pivotal studies in this field reported insulin-producing cells obtained either by EC or IPCS but their generation rate was low and with a poor secretory response to high glucose, probably due to the low differentiation efficiency of protocols employed [165–167]. In 2014 a detailed protocol to generate mature and functional insulin-producing cells from SC was published, describing 7 sequential stages to obtain beta cells able to reverse hyperglycemia in diabetic mice [164]. The 7 stages were defined by endoderm, primitive gut hub, posterior foregut, pancreatic endoderm, pancreatic endocrine precursors, immature beta cells, and maturing beta cells. Veres demonstrated that during these processes only 45% of produced cells are beta-cell [168] since, at each step of the process, a consistent fraction of the cells deviated from the desired path: the consequence of this was the generation of an array of different cell phenotypes such as alfa cells, non-endocrine pancreatic exocrine cells, enterochromaffin cells, and also replicating cells which poses a serious question about malignancy risk. The beta cells demonstrated their functionality when transplanted in diabetic mice in 40 days, showing secretion of C-peptide and insulin in response to glucose. Maturation of the beta cells could be obtained *in vitro* with the administration of different small-molecules and hormones, or *in vivo*, with the transplantation of pancreatic progenitor [169]: it has been established that, *in vivo*, maturation is not related to the pancreas environment since maturation was obtained also after pancreas progenitor transplantation in mice kidney surface [170], thus suggesting that a critical point is a micro vascularization that supplies nutrients and oxygen. In this context, several groups are developing encapsulation devices that allow both substrate supply for beta cells and protection against immune attack; furthermore, encapsulation has the potential to limit the risk of neoplasm formation due to the presence of undifferentiated cells. The preparation of the ideal device should contemplate the biocompatibility of the membrane, the possibility of exposure to blood to allow adequate metabolism for the cells, the adequate release of insulin, and sufficient isolation from immune-competent cells. Thus, major difficulties are related to the finding of an optimal balance between permeability and defense against the host's immune response. Novel cell encapsulation systems are being developed to overcome these problems, and studies in humans are ongoing to evaluate the role of this approach [171]. A different strategy consists of the production of IPC directly from diabetic patients to overcome several obstacles related to the immune response. It has been documented that the production of IPC from skin fibroblasts of T1Ds is reliable [172], and it has been confirmed that these cells are similar to adult beta cells and able to produce insulin in response to glucose variation both *in vitro* and in murine models [173]. Limits of this approach are related to differences intrinsic to patients with T1Ds, with the need to develop different stem cell lines. In conclusion, today the main challenges in developing a beta cell replacement using stem cells are related to 1 the efficient generation of safe and functional insulin-producing cells (pancreatic progenitor or beta cells); 2. the transplantation of cells that do not spark the immune response; 3. conditions that allow adequate nutritional support; 4. the protection from the risk of malignant transformation; 5. a durable normalization of glycemia. However, several progress has been performed in the last decades suggesting that stem cell-based therapy for T1DM could represent the most advanced approach for a definitive cure of T1D.

### Gene therapy

Gene therapy has also been considered to achieve permanent restoration of insulin production [174]: studies in this field confirmed the possibility of obtaining ectopic insulin production from different cells, for example, keratinocytes or fibroblasts [175, 176] using ex vivo gene transfer methods. Using *in vitro* techniques, gene transfer genetically modified cells *in vitro*, then they are transplanted into the subjects: in animal models, this approach allowed a secretion of insulin able to promote glucose uptake and normalize glycemia. *In vivo* gene transfer is performed by viral vectors that modify cells, such hepatocytes, to produce insulin: in murine models, glucose-dependent insulin production by the liver has been demonstrated, with a parallel correction of hyperglycemia [177]. However, gene therapy has some limitations related to risk related to genes chromosomal integration, viral vector safety, and immune response against virus used *in vivo* transfer. Gene therapy could be also applied to other mechanisms involved in overt diabetes progression: *in vivo* gene transfer of antiapoptotic factors demonstrated an increased number of beta cells survival by reducing apoptosis induced by the immune response [178]. Although there are no studies available in humans, the results obtained in animal models suggest a possible role of this approach in the future.

### Prevention of T1D

T1D is an autoimmune disease: with his background in mind, trials have been conducted to halt or slow down the natural history of the disease. Viruses have been considered responsible for the immune response, so vaccination against viruses associated with T1D have been tested [179]. Also, the induction of immune tolerance to beta-cell antigens, such as GAD or insulin, have been explored [180, 181]. None of these studies was successful since they did not delay beta cell destruction [182]. Similar results were obtained with subcutaneous insulin administration [183]. The gut microbiome has a role in immune regulation, and it has been shown a correlation between specific bacterial species and T1D development. Although there is no evidence about the modification in microbiota in the prevention of T1D, this hypothesis could be explored in the future to determine how the gut can modulate immune regulation [184, 185]. Immunosuppressive therapy was also assessed to maintain insulin secretion in the early phase of T1D. Some studies performed in the early 90 showed that the treatment with cyclosporine increased remission rate in new-onset diabetic subjects [186] during 2 years follow up but obvious drug toxicity restrained its use. However, these data suggested that the immunosuppression was able to preserve beta cells from the immune, encouraging studies in this field. For example, in subjects with new-onset diabetes, therapy with a low dose of anti-thymocyte reduced the decline beta-cell function ad improved HbA1c more than subjects treated with placebo [187]. Anti-CD20 monoclonal antibody demonstrated a significant reduction in c peptide decline vs placebo 1 year after drug infusion [188] and also other agents were evaluated with similar results, offering new approaches for the cure shortly [189]. Overall, these data showed that the natural history of type 1 diabetes could be modified, but further studies are necessary to evaluate the long-term effect of immunosuppressive therapy. Other agents were considered for their role in inflammation and immunomodulation [190]. Recently great interest has emerged about the role of vitamin D in the prevention of T1D high-risk subjects [191]. Preclinical studies in mice demonstrated an effect on beta-cell dysfunction and inflammation [192], supported by epidemiological data that demonstrate a correlation between hypovitaminosis D and T1D [193, 194]. Unfortunately, the evidence for this link was inconclusive and further studies are necessary to test such a hypothesis. Omega-3 polyunsaturated fatty acids (O3PUFA) anti-inflammatory role has been explored but there are little data on their effect: in animal models, dietary intervention with O3PUFA reduces inflammatory markers and the incidence of T1D [195]. Epidemiological data [196] suggest a correlation between omega-3 fatty acid intake and the risk of appearance of diabetes-specific autoantibodies. In table 4 ongoing trial regarding type 1 diabetes prevention have been reported.

## Conclusions

**Fig. 2:**
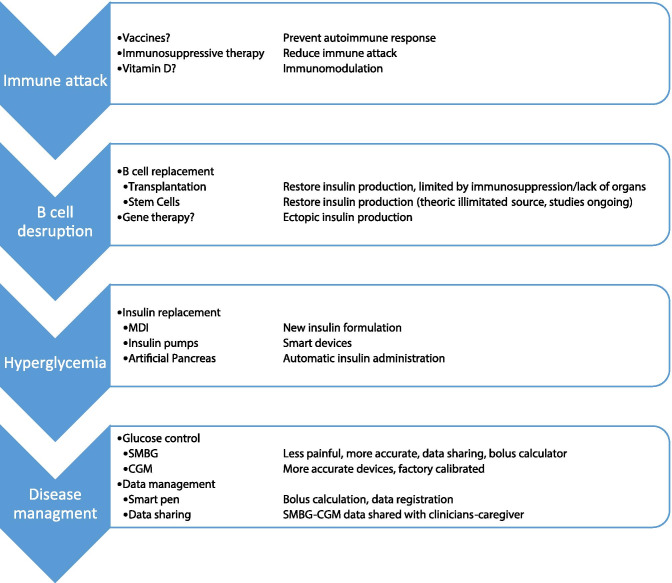
Different steps in diabetes onset and management, actual and future perspectives.. In each phase of diabetes onset different approaches are described in second column and actual and future perspectives are described in third column

Although in the recent year the management of diabetes has dramatically improved, yet the disease has a remarkable impact on subjects with diabetes (Table 5). Particularly in young patients, the burden related to the chronicity and complications calls for new solutions (Table 6). The development of the first models of AP made possible the dream of creating a system able to automatically modify insulin administration through insulin pump based on the values ​​detected by the glucose sensor. AP led to a further improvement of glycemic control with a parallel reduction of burden related to the management of diabetes, especially hypoglycemia. Unfortunately, these systems are not yet fully automated and still require the patient's intervention, especially during the and physical activity. Although the technology can be considered today the most advanced way to manage diabetes, a definitive cure could be obtained only through the biological approaches that guarantee a constant replacement of insulin such as pancreas transplants, and islet cell transplants. In perspective, stem cells, and the possibility of creating new potentially, unlimited beta cells, likely not requiring immunosuppressive therapy, could be finally the cure for diabetes (Fig. 2).

**Table 3:** summary of the meta-analysis regarding artificial pancreas use in the outpatient setting

Meta-Analysis	Population	Number of studies considered	Change in time in range (70–180 mg/dl)	Change in time below range (< 70 mg/dl)
2017 Weisman A, Bai JW, Cardinez M, Kramer CK, Perkins BA [102]	Adults and children	All studies (24) *Single-hormone (22) Bi-hormonal (7) ^#^Adults (10) Pediatric (11)	+ 12·59% (p < 0.0001) + 11.06% (p < 0.0001) + 19.52% (p < 0.0001) + 12.67% (p < 0.0001) + 12.30% (p = 0.0001)	-2.45% (p = 0.003) -1.88% (p = 0.02) -3.78% (p < 0.0001) -1.23% (p = 0.02) -1.58% (p = 0.14)
2018 Bekiari E, Kitsios K, Thabit H, Tauschmann M, Athanasiadou E, Karagiannis T, Haidich AB, Hovorka R, Tsapas A [101]	Adults and children	41 studies 32 Single Hormone 5 Bi-hormonal 4 single hormone and Bi-hormonal system against a control treatment	+ 9.62 (p < 0.001)	-1.49 (p < 0.001)
2019 Karageorgiou V, Papaioannou TG, Bellos I, Alexandraki K, Tentolouris N, Stefanadis C, Chrousos GP, Tousoulis D [103]	Children	25 studies 23 single-hormone 2 Bi-hormonal	+ 11.97% (p = 0.0003)	-0.67% (p = 0.004)

*two comparisons assessed both dual-hormone and single-hormone systems in a three-way crossover design

^#^in three studies, pediatric (≤ 18 years) and adult (> 18 years) patients’ data were entered as separate comparisons in the meta-analysis

**Table 4:** Ongoing study about T1D prevention. In the table is reported a brief description of the trial, date of estimated study completion, population enrolled, and planned outcomes

Study name	Estimated study completation	Description of trial	Population	Outcomes
NCT02620072 Fr1da Insulin Intervention	June 2021	Effect of oral insulin for 12 months in a 24 follow up after last administration	Children from 2 to 12 years Positive for at least two islet autoantibodies	Immune response against insulin Rate of progression to dysglycemia
NCT01773707 CTLA4-Ig (Abatacept)for Prevention of Abnormal Glucose Tolerance and Diabetes in Relatives At -Risk for Type 1	November 2021	Effect of CTLA4-Ig (Abatacept) administered mothly for 1 year	Subjects between 1–45 years with at least two diabetes-related autoantibodiesars	Change from Normal Glucose Tolerance to Abnormal Glucose Tolerance
NCT02605148 TEFA Family Prevention: Glutenfree Diet to Preserve Beta-cell Function (TEFA)	December 2021	Effect of gluten free diet vs normal diet	Subjects between 2 and 50 years with at least one type 1 diabetes-associated autoantibody	Change in first phase insulin response, c-peptide production and glucose metabolism
NCT03428945 Hydroxychloroquine in Individuals At-risk for Type 1 Diabetes Mellitus (TN-22)	August 2024	Effect of Hydroxychloroquine vs placebo	Subjects with more than 3 years with two or more diabetes-related autoantibodies	Changes in glucose tolerance
NCT03182322 PINIT Study: Primary Intranasal Insulin Trial	December 2020	Effect of intranasal administration of insulin for 6 months vs placebo	Children from 1 to 7 years with high genetic risk for T1D	Activation of immune response against insulin
NCT03364868 GPPAD-POInT (Global Platform of Autoimmune Diabetes—Primary Oral Insulin Trial)	January 2025	Effect of daily administration of oral insulin for 3 years vs placebo	Infant between the ages of 4 months and 7 months with a high genetic risk	Development of multiple beta cell autoantibodies Development of diabetes
NCT04014660 Prevention av Autoimmunitet Med Laktobaciller (PAL)	December 2021	Effect of dietary supplement (capsules) containing freeze dried bacteria (active lactobacilli culture) for 12 months vs placebo	Screened persistent positive for any of the auto-antibodies associated with celiac disease (tTGa), type 1 diabetes (IAA, GADA, IA-2A, Zn-T8) and/or thyroid disease (TPOA)	Levels of auto-antibodies

Data available on Clinical Trials.gov

**Table 5:** Actual T1D therapy with advantages and limitations are represented, with future perspectives

	Pro	Cons	Perspectives
MDI	Cost New insulin with more flexibility in administration	Need for multiple injections No data download/sharing	Smart insulin pens
Insulin pump	HbA1c reduction Hypo reduction Complication reduction Increase survival	DKA risk Advance management skills Need for a team with expertise	Automatic devices
SAP Therapy	HBa1c reduction Hypo reduction Improvement on QoL	Alarm fatigue Accuracy Needle Advance management skills Need for a team with expertise	Factory calibrated devices Increased accuracy New sensors
HCL	HbA1c reduction Hypo reduction QoL (?)	Alarms fatigue Advance management skills Need for a team with expertise	FCLC
Pancreas transplantation	Remission of disease Reduction of complication	Immunosuppression Surgical intervention	Stem cells
Islet Transplantation	Remission of disease	Immunosuppression	Islet Encapsulation Stem cells Xenotransplantation

**Table 6:** Diabetes challenges and pharmacology, technology and biology approaches to solve them

Challanges	Pharmacology	Technology		Biology	
		Today	Tomorrow	Today	Tomorrow
Glycemic control	New insulin Adjunctive therapies	Insulin pump CGM CLC	FCLC Bihormonal	Pancreas/islet transplantation	Stem cells Gene therapy
Hypoglycemia	New insulin	CGM CLC	FCLC Bihormonal	Pancreas/islet transplantation	Stem cells Gene therapy
Burden disease-related	-	CGM CLC Smart pen Data sharing	FCLC	Transplantation (limited by immunosuppressant)	Stem cell with no immunosuppressive therapy
Quality of life	(new insulin)	CLC	FCLC	Transplantation (limited by immunosuppressant and surgical intervention)	Stem cell with no immunosuppressive therapy
Prevention of disease	Immunosuppression Vit D?	-	-	-	Gene Therapy
Cure	-	-	-	Transplantation	Stem cell with no immunosuppressive therapy

## Declarations

### Human and animal participants

This article does not contain any studies with human participants or animals performed by any of the authors.
